# *Lactobacillus plantarum* Strains Can Enhance Human Mucosal and Systemic Immunity and Prevent Non-steroidal Anti-inflammatory Drug Induced Reduction in T Regulatory Cells

**DOI:** 10.3389/fimmu.2017.01000

**Published:** 2017-08-23

**Authors:** Paul de Vos, Zlatan Mujagic, Bart J. de Haan, Roland J. Siezen, Peter A. Bron, Marjolein Meijerink, Jerry M. Wells, Ad A. M. Masclee, Mark V. Boekschoten, Marijke M. Faas, Freddy J. Troost

**Affiliations:** ^1^Top Institute Food and Nutrition, Wageningen, Netherlands; ^2^Department of Pathology and Medical Biology, University of Groningen, University Medical Center Groningen, Groningen, Netherlands; ^3^Division of Gastroenterology-Hepatology, Department of Internal Medicine, NUTRIM School for Nutrition, and Translational Research in Metabolism, Maastricht University Medical Center, Maastricht, Netherlands; ^4^Centre for Molecular and Biomolecular Informatics, Radboud University Medical Centre, Nijmegen, Netherlands; ^5^Microbial Bioinformatics, Ede, Netherlands; ^6^NIZO Food Research, Ede, Netherlands; ^7^Department of Host-Microbe Interactomics, Wageningen University, Wageningen, Netherlands; ^8^Department of Human Nutrition, Wageningen University, Wageningen, Netherlands

**Keywords:** intestinal mucosal immunity, adaptive immunity, *Lactobacillus plantarum*, indomethacin, non-steroidal anti-inflammatory drug, probiotics

## Abstract

Orally ingested bacteria interact with intestinal mucosa and may impact immunity. However, insights in mechanisms involved are limited. In this randomized placebo-controlled cross-over trial, healthy human subjects were given *Lactobacillus plantarum* supplementation (strain TIFN101, CIP104448, or WCFS1) or placebo for 7 days. To determine whether *L. plantarum* can enhance immune response, we compared the effects of three stains on systemic and gut mucosal immunity, by among others assessing memory responses against tetanus toxoid (TT)-antigen, and mucosal gene transcription, in human volunteers during induction of mild immune stressor in the intestine, by giving a commonly used enteropathic drug, indomethacin [non-steroidal anti-inflammatory drug (NSAID)]. Systemic effects of the interventions were studies in peripheral blood samples. NSAID was found to induce a reduction in serum CD4^+^/Foxp3 regulatory cells, which was prevented by *L. plantarum* TIFN101. T-cell polarization experiments showed *L. plantarum* TIFN101 to enhance responses against TT-antigen, which indicates stimulation of memory responses by this strain. Cell extracts of the specific *L. plantarum* strains provoked responses after WCFS1 and TIFN101 consumption, indicating stimulation of immune responses against the specific bacteria. Mucosal immunomodulatory effects were studied in duodenal biopsies. In small intestinal mucosa, TIFN101 upregulated genes associated with maintenance of T- and B-cell function and antigen presentation. Furthermore, *L. plantarum* TIFN101 and WCFS1 downregulated immunological pathways involved in antigen presentation and shared downregulation of snoRNAs, which may suggest cellular destabilization, but may also be an indicator of tissue repair. Full sequencing of the *L. plantarum* strains revealed possible gene clusters that might be responsible for the differential biological effects of the bacteria on host immunity. In conclusion, the impact of oral consumption *L. plantarum* on host immunity is strain dependent and involves responses against bacterial cell components. Some strains may enhance specific responses against pathogens by enhancing antigen presentation and leukocyte maintenance in mucosa. In future studies and clinical settings, caution should be taken in selecting beneficial bacteria as closely related strains can have different effects. Our data show that specific bacterial strains can prevent immune stress induced by commonly consumed painkillers such as NSAID and can have enhancing beneficial effects on immunity of consumers by stimulating antigen presentation and memory responses.

## Introduction

Commensal Lactobacilli species may play an active role in intestinal immune homeostasis (1–9). Lactobacilli likely regulate immune cells *via* the interaction of bacterial cell-wall components or secreted bacterial products with immune or epithelial cells in human gut mucosa (8, 10, 11). These interactions seem to contribute to more than just tolerance to the beneficial microbes. As demonstrated in several vaccination studies Lactobacilli, such as *plantarum* strains derived from WCFS1, have a positive impact on immune responses (12–18). Furthermore, these bacteria can activate tolerogenic cellular pathways in human intestinal mucosal cells (3, 5, 10).

In previous studies, we demonstrated that *Lactobacillus plantarum*, a member of lactic acid bacteria with a “generally recognized as safe” status (3, 19), had different effects on human dendritic and peripheral blood mononuclear cells (10, 11). Using comparative genomic hybridization, we identified a number of bacteriocins and cell-wall components involved in the glycosylation of cell wall teichoic acids associated with these differential effects (8, 10). The differential expression of *L. plantarum* genes may contribute to the observed differences in activation of toll-like receptor (TLR) 2–4 and CD14 antigens in the host (10). As a consequence of differences in TLR-binding dendritic cells *L. plantarum* strains induce alterations in production of the pro-inflammatory cytokine IL-12 and the regulatory cytokine IL-10 (10). The immunomodulatory effects of *L. plantarum* may benefit human gut homeostasis, in particular in case of pathogenic or pharmacological induced mucosal stress. Non-steroidal anti-inflammatory drugs (NSAIDs), which are commonly used painkillers, are well known for their negative side effects on gut mucosal integrity and immunity, which are mediated through inhibition of cyclooxygenase and subsequent prostaglandin deficiency. But these effects have also been shown to be modulated by gut microbiota *via* among others TLR4 signaling (20).

The current clinical trial has been conducted to study the effects of three *L. plantarum* strains on immunity and intestinal barrier function. The findings regarding immune responses are presented in the current report, while the results on gut barrier function have been published previously (2). We have shown that small intestinal permeability of healthy volunteers increased, indicating gut barrier dysfunction, after administration of indomethacin (a NSAID) which could not be reversed by intake of *L. plantarum* strains. However, one of the strains, *i.e*., *L. plantarum* TIFN101, did demonstrate profound effects on mucosal gene transcription related to mucosal structure, while the findings regarding the modulation of these processes by *L. plantarum* WCFS1 and CIP4448 were more moderate. This indicates strain-dependent modulation of gut barrier function by the tested *L. plantarum* strains (2).

To investigate mechanism by which *L. plantarum* may influence *in vivo* human mucosal and systemic immune activity under mild mucosal stress conditions such as intake of NSAIDs, we conducted a randomized double-blind placebo-controlled cross-over human trial. Three *L. plantarum* strains were selected for their different immunomodulating effect *in vitro* (3, 10, 11). The three strains have different effects on TLRs signaling and differently stimulate cytokine production *in vitro* in immune cells (3, 10, 11). Healthy volunteers received the NSAID indomethacin, which induces mild and reversible damage to the gastrointestinal lining (21–24). Study participants subsequently consumed one of the three *L. plantarum* strains or placebo for a week. Polarized T-cell responses in the peripheral circulation, as well as the responses after re-stimulation with cell extract of *L. plantarum* strains, superantigen, or tetanus were studied to determine whether these bacteria could enhance systemic immunity against one of the stimuli. Duodenal biopsies were taken and used to study the effect of the strains on mucosal gene regulation. Furthermore, the bacterial strains were sequenced and annotated to identify putative gene clusters associated with the differential responses induced by the probiotics.

## Materials and Methods

This study was approved by the University Hospital Maastricht Ethical Committee and has been registered in the US National Library of Medicine (*NCT01456767*).^1^ A more detailed description of the trial design has been published previously (2).

### Study Design and Study Participants

Ten healthy volunteers, seven females and three males (26.3 ± 10.1 years, BMI of 21.8 ± 2.40 kg/m^2^), without a history of gastrointestinal symptoms and free of medication, were tested on four separate occasions in a randomized cross-over design. Four intervention periods included the 7-day intake of *L. plantarum* strains WCFS1 (WCFS), CIP104448 (CIP48), or TIFN101, or placebo. Before and after the intervention, blood samples were taken to study the effect of *L. plantarum* consumption on T-cell polarization. Furthermore, at day 7, duodenal biopsies were taken for whole-genome expression microarrays. No side effects or complications were reported.

All included subjects gave their written informed consent. The healthy volunteers consumed their habitual diet and kept a gastrointestinal symptoms diary. The night before the start day, the volunteers ingested 75 mg of the NSAID indomethacin. On the start day, the volunteers ingested another dosage of 50 mg indomethacin to conform to a previously established protocol to establish mild gastrointestinal mucosal stress (25, 26). Subsequently, the volunteers consumed the bacterium or placebo supplements for a period of 7 days during breakfast and during dinner. The vials containing bacteria or placebo were non-transparent. On the seventh day, blood samples were taken again and tissue samples were obtained from the horizontal part of the duodenum by standard flexible gastroduodenoscopy at approximately 15 cm distal to the pylorus. The duodenal mucosa was chosen as the duodenum is readily accessible for sampling. The interventions were performed with an interval of 4 weeks to allow a wash-out period and also healing of the biopsy-sampling region.

### Bacterial Strains and Growth Conditions

Three *L. plantarum* strains form the NIZO strain-collection were selected: WCFS1, CIP104448, and TIFN101. In previous studies TIFN101 has been referred to as CIP104450 (11, 27). The bacteria were cultured at 37°C in man, rogosa, and sharpe medium (Merck). Detailed protocols for culturing, harvesting, freeze drying, storing, and viability determination of *Lactobacillus* species have been published (1). Maltodextrin and glucose were added to a final concentration of 20 and 2% (wt/vol), respectively, to obtain bacterial preparations (WCFS1, 2.6 × 10^9^ CFU; CIP104448, 2.4 × 10^9^ CFU; and TIFN101, 5.6 × 10^9^ CFU); placebo controls contained the two sugars.

### Cell Staining

Blood of study participants was collected in EDTA-containing tubes and processed for fluorescence-activated cell sorting (FACS) analysis (Table S1 in Supplementary Material). Isotype controls were used at the same dilution as the antibody.

To study T-cell polarization, 200 µl of blood was diluted with 200 µl of RPMI1640 supplemented with heat-inactivated 10% fetal calf serum (FCS) and incubated with either phorbol-myristate-acetate (PMA; 80 nM Sigma-Aldrich, Steinheim, Germany) and 2 nM calcium ionophore (Ca-Io; Sigma-Aldrich) (4 h), *Staphylococcus aureus*-enterotoxin B (SEB) (5 µg/ml Sigma, Deisenhofen, Germany) (24 h), tetanus toxoid (TT; 1.5 Lf/ml, RIVM, Bilthoven, The Netherlands) (24 h), or bacterial lysates (30 µg/ml) (24 h). Stimulation with bacterial lysates was performed 1 week after treatment with one of the three strains. After stimulation, red blood cells were lysed with ammonium chloride, washed (PBS with 2% FCS), and incubated with different antibody cocktails.

To stain for T-cells and T-cell subsets, cells were incubated with an antibody cocktail consisting of anti-CD3, anti-CD8, and anti-CD45RO for 30 min in the dark on ice. After washing with buffer, cells were incubated with streptavidin-Pacific Orange (1:100 Invitrogen) for 15 min on ice. After washing and centrifugation, pelleted cells were resuspended in Fix/Perm solution (eBioscience) for 45 min on ice. After washing in Perm-solution cells were incubated in mouse-serum for 15 min, followed by incubation with the cytokine antibody mix (anti-IL-4, anti-IFNγ, anti-IL-17, and anti-IL-21) or an isotype control mix for 30 min on ice. Cells were then washed with Perm solution (three times), resuspended in wash-buffer and measured by flow-cytometry.

For staining NK-cells, cells were incubated with an antibody cocktail consisting of anti-CD3, anti-CD16, anti-CD56, anti-CD335, and anti-CD161 (NK-cell staining), or with an isotype control cocktail for NK-cells consisting of anti-CD3, anti-CD16, anti-CD56, and isotype controls for anti-CD335 and CD161 for 30 min in the dark on ice. After washing with washing buffer, cells were fixed in FACS-lysis solution (BD Biosciences) for 30 min on ice, washed and analyzed by flow-cytometry.

### RNA Isolation and Microarray

Total RNA was isolated from duodenal biopsy samples of the study participants using Trizol reagent (1 ml) (Invitrogen, Breda, The Netherlands). RNA was purified using the Qiagen RNeasy Micro kit (Qiagen, The Netherlands) and quantified on a NanoDrop ND-1000 spectrophotometer (Isogen Life Science, The Netherlands). RNA quality was confirmed using an Agilent 2100 bioanalyzer (Agilent Technologies, The Netherlands). RNA was judged suitable for array-hybridization only if samples exhibited intact bands corresponding to 18S and 28S ribosomal subunits and displayed no chromosomal peaks or RNA-degradation products.

Total RNA (100 ng) was used for whole transcript cDNA synthesis using the Ambion WT expression kit (Life Technologies, The Netherlands) and was subsequently labeled using the Affymetrix GeneChip WT Terminal Labeling Kit (Affymetrix, Santa Clara, CA, USA). Samples were hybridized to human whole genome Affymetrix GeneChip Human Gene 1.1 ST arrays, washed, stained, and scanned on an Affymetrix GeneTitan instrument. Details on array handling can be found in the Affymetrix GeneTitan Instrument User Guide for Expression Array Plates (P/N 702933 Rev.2).

### Duodenal Mucosa Microarray Data Analysis

Microarray analysis was performed utilizing MADMAX for statistical analysis (28). All arrays met the quality criteria. The probes on the Human Gene 1.1 ST arrays were redefined according to Dai et al. (29) based on the NCBI Entrez database (CDF version 15.1). In this way, the Human Gene 1.1 ST array targets 19,682 unique genes. Normalized expression values were obtained from the raw intensity values by using the robust multi-array analysis preprocessing algorithm available in the AffyPLM library using default settings (30). Microarray data were filtered and probe sets with at least five probes and expression values higher than 20 on at least five arrays with an interquartile range (IQR) >0.2 (log2 scale) across all samples were selected for further statistical analysis. In addition, an IQR cut-off of 0.2 was used to filter out genes that showed no variation between the conditions. Differentially expressed genes were identified using linear models, applying moderated t-statistics that implemented empirical Bayes regularization of SEs in the library limma (31). The moderated *t*-statistic was extended by a Bayesian hierarchical model to define an intensity-based moderated t-statistic to adjust for independence of variances relative to the degree of identity and relation between variance and signal intensity (32). Genes were defined as significantly changed when the *p*-value was <0.05 for pairwise comparisons. The datasets generated from microarray profiling experiments have been deposited to the publicly accessible database repository Gene Expression Omnibus (GSE74988).

### Pathway Analysis

Gene set enrichment analysis (GSEA^2^) was performed using MADMAX and gene sets with a false discovery rate <0.2 were considered significantly enriched. GSEA takes into account the broader context in which gene products function, namely in physically interacting networks such as biochemical, metabolic, or signal transduction routes, and has the advantage that it is unbiased because no gene selection step is used. Possible transcription factors were identified using upstream regulator analysis in ingenuity pathway analysis (IPA; Ingenuity Systems, Redwood City, CA, USA).

### Bacterial Genome Sequencing and Annotation

For DNA preparation, bacteria were pelleted, washed, and resuspended in TES buffer (*N*-[tris(hydroxymethyl)methyl]-2-aminoet hanesulfonic acid). Cells were lysed with lysozyme (360 mg/ml) and mutanolysin (140 U/ml) by incubation for 2 h at 37°C. Subsequently, 300 µl water and 80 µl of a 20% SDS solution were added. The DNA extraction was done using phenol/chloroform (3×). DNA was precipitated with isopropanol and washed with 70% ethanol. Samples were treated with 100 µg/ml RNAse (Sigma) for 1 h at 37°C. DNA paired-end libraries with barcoding were made and sequenced using Illumina technology (Baseclear Leiden). The genome sequences of *L. plantarum* strains CIP104448 and TIFN 101 have been deposited in NCBI/GenBank under accession numbers JSUW00000000 and JSUX00000000, respectively. The contig sequences were submitted to the RAST automatic annotation server,^3^ which provided ORF calling and automatic annotation. The annotated contigs of CIP48 and TIFN101 were ordered by comparing them to the circular template genome of *L. plantarum* WCFS1 (33), and comparing them to each other. Contigs/genes that did not match to the WCFS1 genome were annotated in more detail using BLASTP^4^ and InterProscan.^5^ Ortholog groups (OGs) in the three genomes were determined using OrthoMCL.^6^

### Statistics

Flow-cytometry results are expressed as the mean ± SEM. Normal distribution was confirmed by the Kolmogorov–Smirnov test. The two-sided Student’s *t*-test was used for changes in immune-cell populations after *L. plantarum* treatment. Gene expression data are depicted as the median (range). The two-sided Mann–Whitney *U*-test was used to determine changes in expression profiles after *L. plantarum* treatment *in vivo*. A *p*-value <0.05 was considered statistically significant.

## Results

### Cell Frequencies and T-Cell Polarization after Treatment with *L. plantarum* Strains

**Figure 1:**
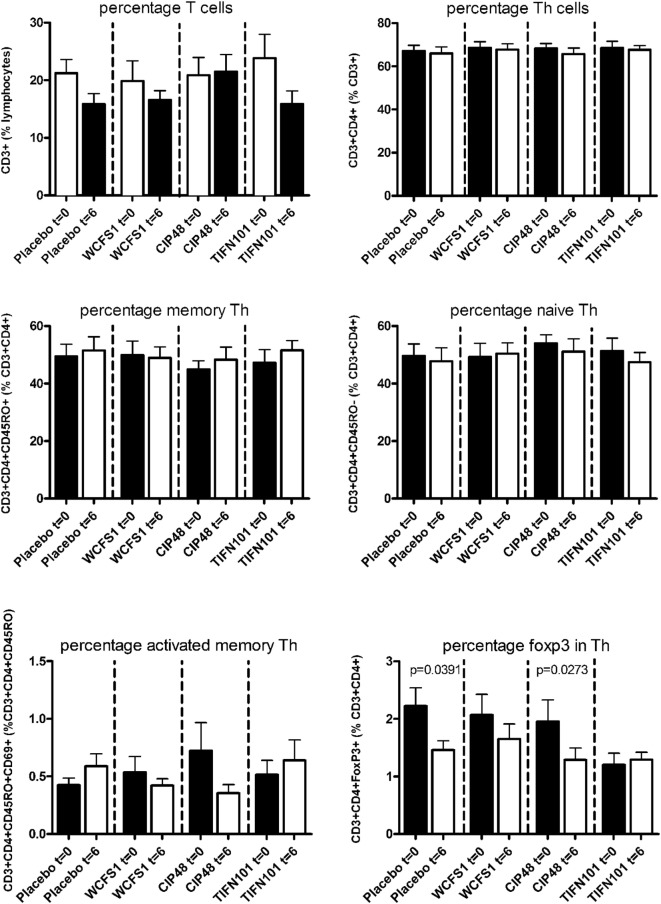
Effects of three *Lactobacillus plantarum* strains on the frequency of CD4^+^ T-cell populations in systemic circulation. Data are expressed as mean ± SEM. Statistical significant differences were determined by using two-sided Student’s *t*-tests. A *p*-value <0.05 was considered statistically significant. A *p*-value <0.1 was considered a statistical trend.

**Figure 2:**
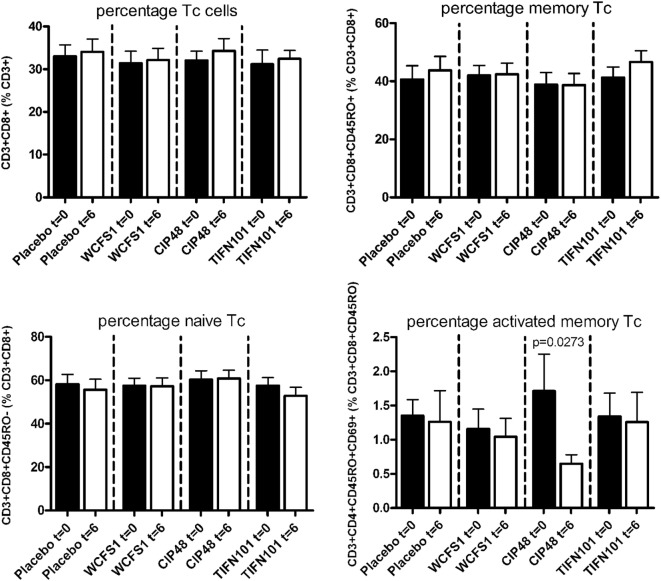
Effects of three *Lactobacillus plantarum* strains on the frequency of CD8^+^ T-cell populations in systemic circulation. Data are expressed as mean ± SEM. Statistical significant differences were determined by using two-sided Student’s *t*-tests. A *p*-value <0.05 was considered statistically significant. A *p*-value <0.1 was considered a statistical trend.

In peripheral blood samples, we did not observe differences in the frequencies of total CD3^+^ cells, CD3^+^CD4^+^ cells (naïve or memory), or the CD3^+^CD4^+^ activated memory cells after treatment with any of the *L. plantarum* strains. However, the percentage of CD4^+^Foxp3^+^ cells was significantly decreased following placebo and CIP48 treatment, but not after TIFN101 treatment (Figure 1). Moreover, although we did not observe any effect with indomethacin treatment on CD3^+^CD8^+^ (naïve and memory) cells, activated memory-cells exhibited a statistically significantly decrease after CIP48 treatment (*p* < 0.05) (Figure 2).

Treatment did not influence percentages of NK-cells or NKT-cells. There was also no change in the percentages of the NK-cell subtypes (i.e., CD56^high^ and CD56^dim^); and the expression of CD161 (KLRB1), mediating cytotoxicity (33, 34), was not affected either by the *L. plantarum* treatments (results not shown).

T-cell polarization was studied after three types of T-cell stimulation: (i) non-specific polyclonal stimulation with PMA/Ca^2+^ or superantigen *Staphylococcus aureus*-enterotoxin B (SEB) to study whether the total responsiveness was influenced by *L. plantarum* treatment, (ii) stimulation with a previously administered vaccine-antigen (TT) to study stimulation of specific memory responses, and (iii) stimulation with cell extracts of the specific *L. plantarum* strains in order to investigate whether specific immune responses against the *L. plantarum* were stimulated.

**Figure 3:**
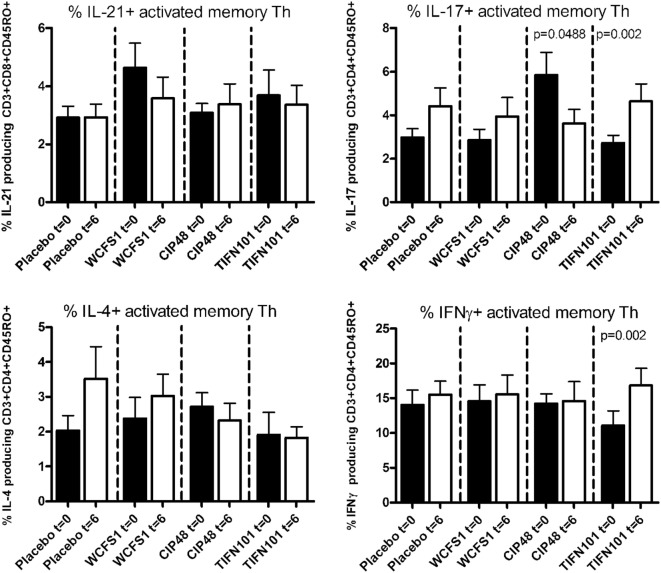
Effects of three *Lactobacillus plantarum* strains on the frequency of IL-21, IL-17, IL-4, and IFN-γ-producing *Staphylococcus aureus* enterotoxin B superantigen (SEB)-stimulated memory-CD45RO^+^ Th cells. Data are expressed as mean ± SEM. Statistical significant differences were determined by using two-sided Student’s *t*-tests. A *p*-value <0.05 was considered statistically significant. A *p*-value <0.1 was considered a statistical trend.

After non-specific stimulation with PMA/Ca-ionophore or SEB, we quantified the percentage of IFNγ, IL-4, IL-17, or IL-21 positive Th cells and memory Th cells. Treatment with placebo or the administered *L. plantarum* strains did not influence cytokine production of the total population of Th cells or of Th memory cells after non-specific stimulation with PMA/Ca-ionophore. Although no differences were found after SEB-stimulation in cytokine production by the total Th cell population after the three *L. plantarum* treatments (results not shown), we did observe differences in cytokine production of the Th memory cells after *L. plantarum* treatment. After stimulation with SEB, we observed a decreased percentage of IL-17-producing activated memory Th cells following treatment with CIP48 and an increased percentage of IL-17-producing activated memory Th cells after treatment with TIFN101 (Figure 3). Moreover, the percentage of IFNγ-producing activated memory Th cells was also increased after TIFN101 treatment (Figure 3).

**Figure 4:**
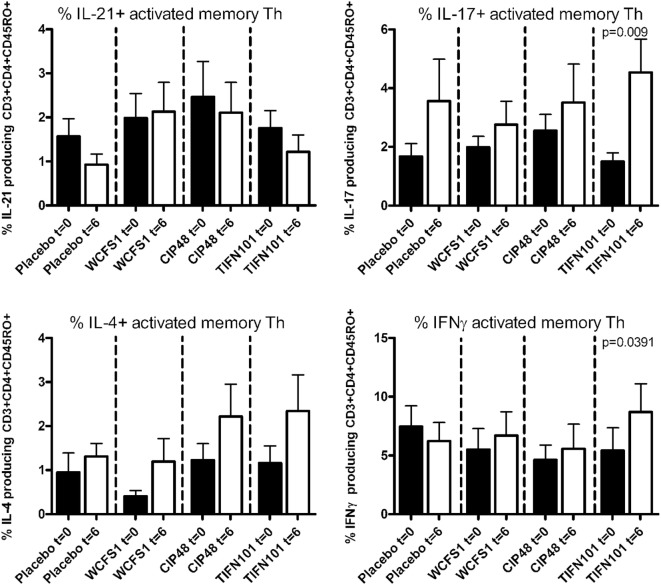
Effects of three *Lactobacillus plantarum* strains on the frequency of IL-21, IL-17, IL-4, and IFN-γ-producing tetanus toxoid-stimulated memory-CD45RO^+^ Th cells. Data are expressed as mean ± SEM. Statistical significant differences were determined by using two-sided Student’s *t*-tests. A *p*-value <0.05 was considered statistically significant. A *p*-value <0.1 was considered a statistical trend.

Treatment with *L. plantarum* strains also modulated cytokine production following a more specific stimulation by TT (Figure 4). After TIFN101 treatment, the percentage IL-17 and the percentage IFN-γ-producing memory Th cells were significantly increased, while no effect on cytokine production by memory Th cells after TT-stimulation was observed with the other *L. plantarum* strains.

**Figure 5:**
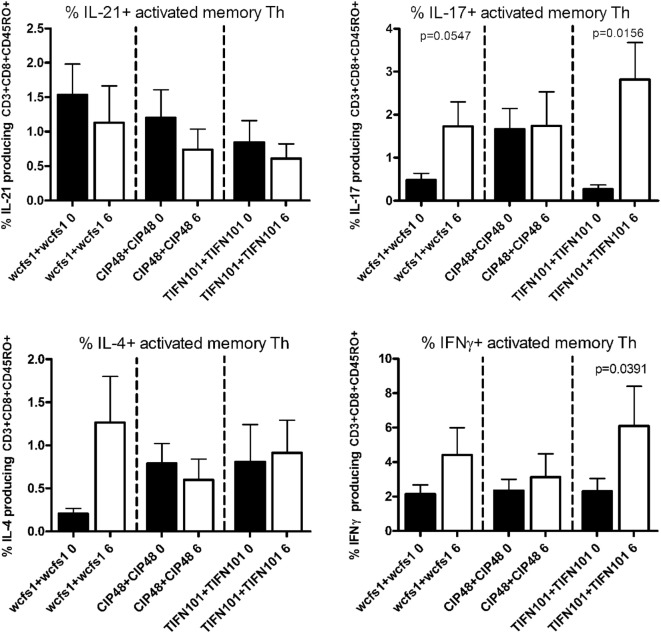
Effects of three *Lactobacillus plantarum* strains on the frequency of IL-21, IL-17, IL-4, and IFN-γ-producing memory-CD45RO^+^ Th cells stimulated with bacterial cell extracts, matched to the strain consumed. Data are expressed as mean ± SEM. Statistical significant differences were determined by using two-sided Student’s *t*-tests. A *p*-value <0.05 was considered statistically significant. A *p*-value <0.1 was considered a statistical trend.

Finally, we stimulated human blood samples with cell extracts of the *L. plantarum* strain that the volunteers had consumed in the study (Figure 5). We observed that subjects, who were treated with WCFS, showed a trend toward an increased IL-17 response after stimulation with WCFS cell extracts. Other cytokines were not affected after this treatment. There were no differences in cytokine production in subjects treated with CIP48, when their blood samples were stimulated with CIP48 cell extract. When subjects were treated with TIFN101, their activated memory cells exhibited increased IL-17 and IFN-γ-production following stimulation with TIFN101 cell extract.

### Transcriptional Response in Duodenal Mucosa upon Exposure to *L. plantarum* Strains

**Figure 6:**
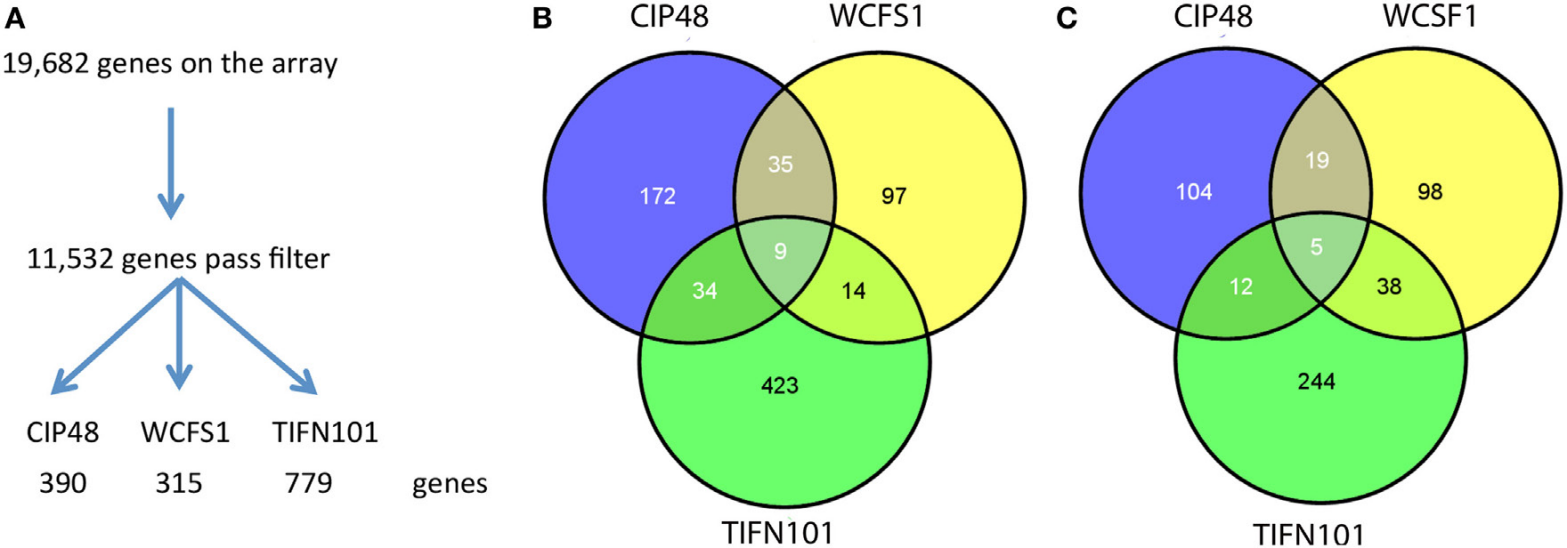
Flowchart of the microarray analysis **(A)** and the number of unique genes regulated in human intestinal biopsies after consumption of three different *Lactobacillus plantarum* strains [*L. plantarum* WCFS1 (WCFS), *L. plantarum* CIP104448 (CIP48), *L. plantarum* TIFN101 (TIFN101)]. Intensity >20 on at least five arrays, interquartile range >0.2, at least seven probes per gene. Venn diagrams of the number of upregulated **(B)** and downregulated **(C)** genes in the intestinal biopsies after consumption of *L. plantarum* and indomethacin.

Differential gene expression profiles were found in the stressed intestinal mucosa of the subjects consuming each bacterial strain; 315 genes were differentially regulated with *L. plantarum* WCFS, 390 with CIP48, and 779 with TIFN101, as compared to the placebo intervention (Figure 6A). Of these genes, the different bacterial strains shared only relatively small numbers of upregulated and downregulated genes (Figures 6B,C, respectively). Shared genes were mainly involved in general cellular functions and metabolism.

The 10 genes that were most highly up- or downregulated are listed in Tables 1–3. Of the most highly induced genes after TIFN101 consumption 80% are related to immunity: immunoglobulin lambda variable 6–57, putative V-set and immunoglobulin domain-containing protein 6-like, immunoglobulin lambda variable 7–46, interferon regulatory factor 4, GDNF family, CD27, CD79a, and plasminogen activator. WCFS and TIFN101 shared the downregulation of six small nucleolar RNAs, i.e., snoRNA (H)C/D(ACA) box 6, 14b, 53, 57, 60, 388, while CIP48 had a complete different profile of up and downregulation.

**Table 1:** *Lactobacillus plantarum* WCFS1 (WCSF): 10 most upregulated and downregulated genes.

	Gene name	IBMT *p*-value	Mean fold versus placebo
**Top 10: upregulated genes placebo versus WCFS**			
Kinesin family member 20B	KIF20B	0.01	1.34
microRNA 186	MIR186	0.03	1.31
Guanylate cyclase activator 2A (guanylin)	GUCA2A	0.04	1.31
Integrin, alpha 4 (antigen CD49D, alpha 4 subunit of VLA-4 receptor)	ITGA4	0.01	1.31
Centromere protein E, 312 kDa	CENPE	0.05	1.30
Putative homeodomain transcription factor 1	PHTF1	0.00	1.30
Spindle and kinetochore-associated complex subunit 2	SKA2	0.01	1.29
Killer cell lectin-like receptor subfamily D, member 1	KLRD1	0.00	1.29
Gamma-aminobutyric acid A receptor, alpha 2	GABRA2	0.02	1.27
Retinitis pigmentosa GTPase regulator	RPGR	0.01	1.27
**Top 10: downregulated genes placebo versus WCFS**			
Small nucleolar RNA, H/ACA box 16A	SNORA16A	0.02	−1.35
Small nucleolar RNA, C/D box 53	SNORD53	0.04	−1.36
Contactin 3 (plasmacytoma associated)	CNTN3	0.00	−1.38
Small nucleolar RNA, C/D box 6	SNORD6	0.04	−1.39
Small nucleolar RNA, H/ACA box 57	SNORA57	0.00	−1.44
Small nucleolar RNA, H/ACA box 60	SNORA60	0.00	−1.44
Small nucleolar RNA, H/ACA box 14A	SNORA14A	0.03	−1.49
Small nucleolar RNA, H/ACA box 38B (retrotransposed)	SNORA38B	0.01	−1.58
Small nucleolar RNA, H/ACA box 14B	SNORA14B	0.01	−1.58
Small Cajal body-specific RNA 4	SNORA16A	0.01	−1.72

**Table 2:** *Lactobacillus plantarum* CIP104448 (CIP48): 10 most upregulated and downregulated genes.

	Gene name	IBMT *p*-value	Mean fold versus placebo
**Top 10: upregulated genes placebo versus CIP48**			
Coiled-coil domain containing 59	CCDC59	0.01	1.33
Aldehyde dehydrogenase 1 family, member L2	ALDH1L2	0.03	1.32
KIAA0125	KIAA0125	0.00	1.31
Phospholipase C, beta 4	PLCB4	0.01	1.31
Coiled-coil domain containing 102B	CCDC102B	0.04	1.29
RAS guanyl releasing protein 3 (calcium and DAG-regulated)	RASGRP3	0.03	1.28
Peptidase domain containing associated with muscle regeneration 1	PAMR1	0.00	1.28
DEP domain containing 1	DEPDC1	0.01	1.28
Phospholipase A2, group IIA (platelets, synovial fluid)	PLA2G2A	0.02	1.28
Heparan sulfate (glucosamine) 3-O-sulfotransferase 3B1	HS3ST3B1	0.04	1.28
**Top 10: downregulated genes placebo versus CIP48**			
Potassium channel, subfamily K, member 15	KCNK15	0.05	−1.33
Transient receptor potential cation channel, subfamily V, member 6	TRPV6	0.01	−1.33
Long intergenic non-protein coding RNA 282	LINC00282	0.03	−1.35
Ephrin-A1	EFNA1	0.02	−1.38
Matrix metallopeptidase 10 (stromelysin 2)	MMP10	0.05	−1.41
Angiopoietin-like 4	ANGPTL4	0.03	−1.47
Heme oxygenase (decycling) 1	HMOX1	0.00	−1.50
Nuclear factor, interleukin 3 regulated	NFIL3	0.01	−1.52
Major facilitator superfamily domain containing 2A	MFSD2A	0.02	−1.59
Glucose-6-phosphatase, catalytic subunit	G6PC	0.02	−1.65

**Table 3:** *Lactobacillus plantarum* TIFN101 (TIFN101): 10 most upregulated and downregulated genes.

	Gene name	IBMT *p*-value	Mean fold versus placebo
**Top 10: upregulated genes placebo versus TIFN101**			
Immunoglobulin lambda variable 6–57	IGLV6-57	0.01	1.63
Putative V-set and immunoglobulin domain-containing protein 6-like	LOC642131	0.00	1.55
Immunoglobulin lambda variable 7–46 (gene/pseudogene)	IGLV7-46	0.04	1.48
Heparan sulfate (glucosamine) 3-O-sulfotransferase 3B1	HS3ST3B1	0.00	1.41
Interferon regulatory factor 4	IRF4	0.00	1.40
GDNF family receptor alpha 2	GFRA2	0.00	1.40
CD27 molecule	CD27	0.01	1.40
CD79a molecule, immunoglobulin-associated alpha	CD79A	0.03	1.38
Plasminogen activator, tissue	PLAT	0.00	1.37
Der1-like domain family, member 3	DERL3	0.00	1.37
**Top 10: downregulated genes placebo versus TIFN101**			
Small nucleolar RNA, H/ACA box 38B (retrotransposed)	SNORA38B	0.04	−1.40
Small nucleolar RNA, H/ACA box 21	SNORA21	0.03	−1.40
Small nucleolar RNA, H/ACA box 60	SNORA60	0.01	−1.41
Small nucleolar RNA, C/D box 53	SNORD53	0.01	−1.43
Ephrin-A1	EFNA1	0.00	−1.45
Small nucleolar RNA, C/D box 6	SNORD6	0.02	−1.46
Small nucleolar RNA, H/ACA box 57	SNORA57	0.00	−1.47
Small nucleolar RNA, H/ACA box 14B	SNORA14B	0.00	−1.73
Family with sequence similarity 5, member C	FAM5C	0.03	−1.97
Small Cajal body-specific RNA 4	SCARNA4	0.00	−2.00

**Figure 7:**
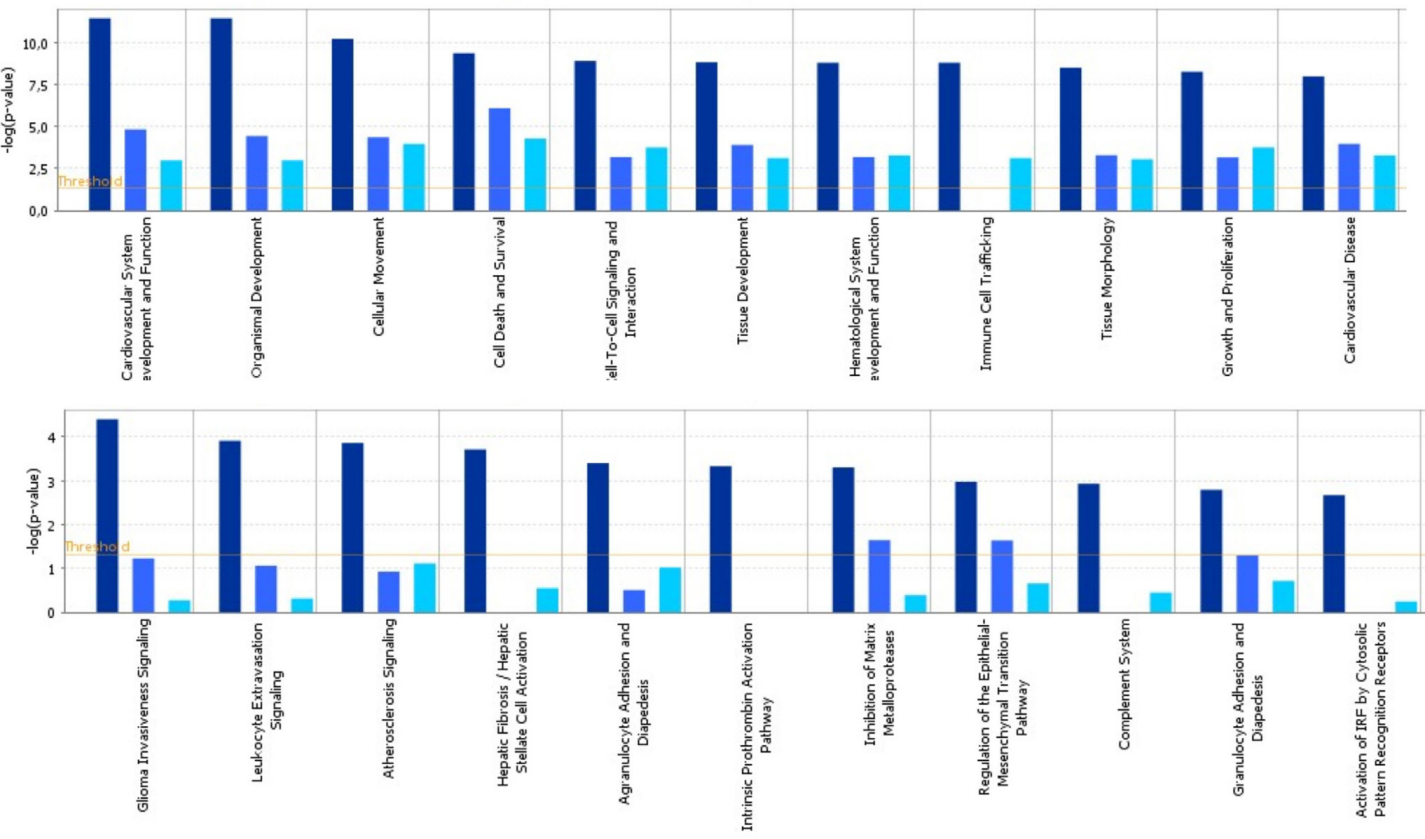
Cellular pathways are significantly more modulated after consumption of either *Lactobacillus plantarum* TIFN101 (dark blue, left column) than after consumption of *L. plantarum* CIP104448 (light blue, middle column) or *L. plantarum* WCFS1 (cyan, right column). Statistical significance of pathway modulation was calculated *via* a right-tailed Fisher’s Exact test in ingenuity pathway analysis and represented as −log (*p*-value); −log values exceeding the depicted threshold were significant (*p* < 0.05).

**Figure 8:**
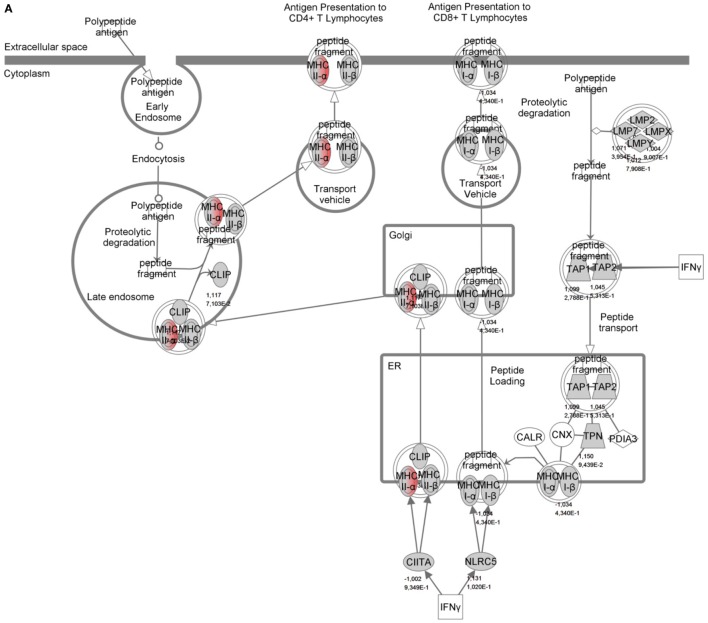

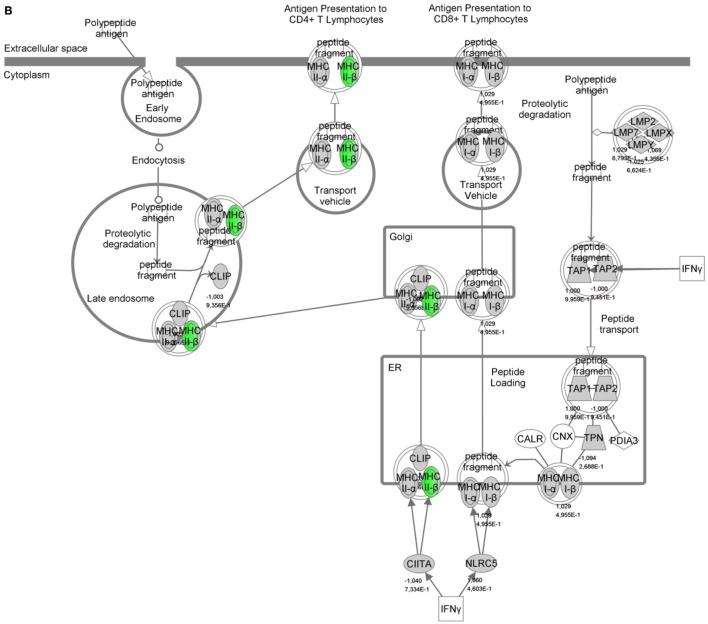

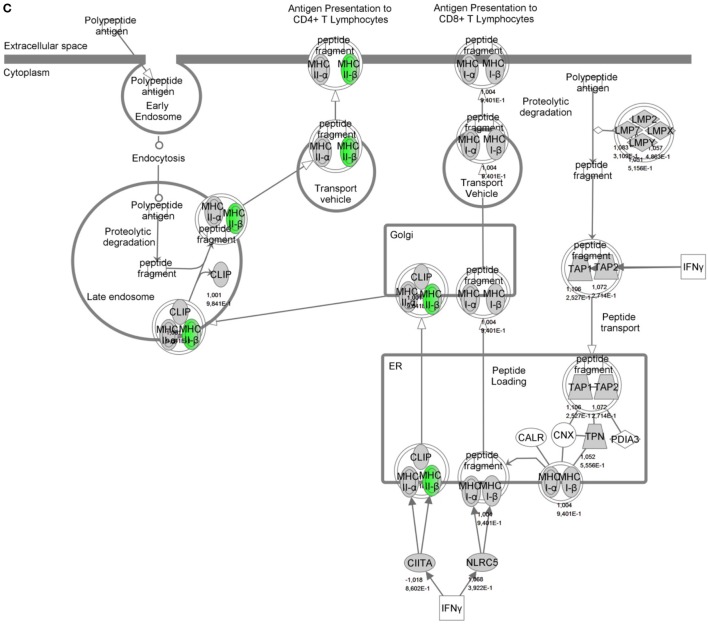
Regulation of genes involved in antigen presentation in non-steroidal anti-inflammatory drug-stressed intestine after consumption of either *Lactobacillus plantarum* TIFN101 **(A)**, *L. plantarum* CIP104448 **(B)**, or *L. plantarum* WCFS1 **(C)**. Red indicates upregulation while green depicts downregulation of the specific gene. TIFN101 upregulated MHC-IIα while CIP48 and WCFS1 downregulated MHC-IIβ.

**Figure 9:**
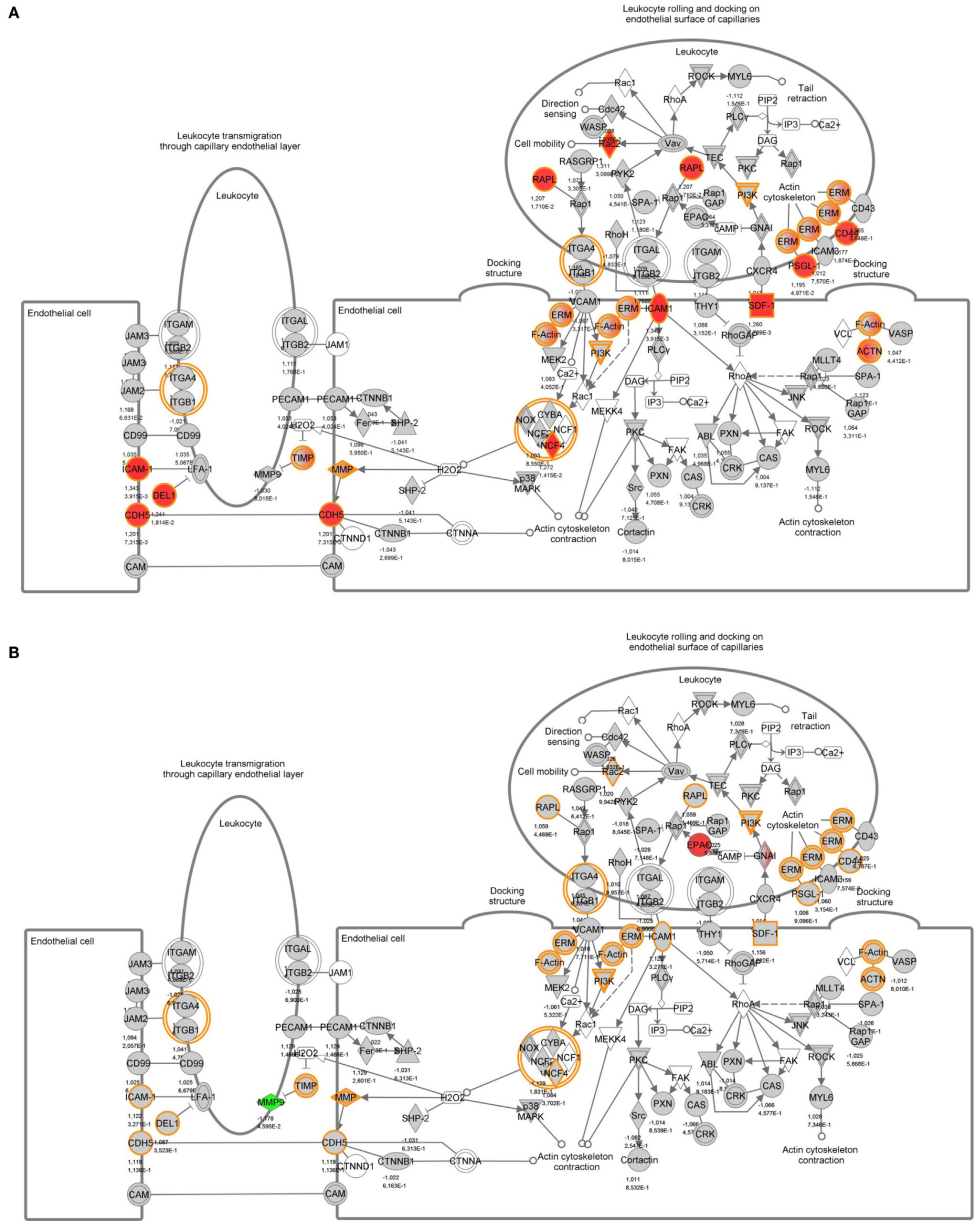

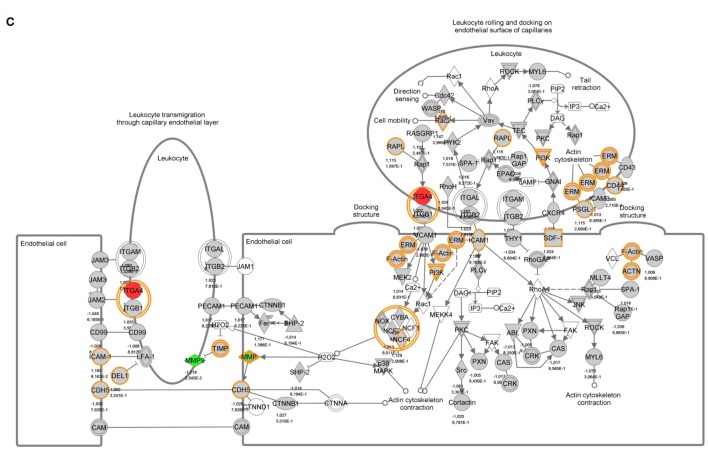
Regulation of genes involved in leukocyte extravasation pathways in non-steroidal anti-inflammatory drug-stressed intestine after consumption of either *Lactobacillus plantarum* TIFN101 **(A)**, *L. plantarum* CIP104448 **(B)**, or *L. plantarum* WCFS1 **(C)**. Red indicates upregulation while green depicts downregulation of the specific gene.

To identify specific transcription factors and to identify pathways regulated by the strains, IPA was performed (Figure 7). TIFN101 induced more changes than CIP48 and WCSF1 in the NSAID-stressed intestine. The most significant set of target-genes in the TIFN101 group were immune response-related genes. TIFN101 upregulated MHC-IIα, while CIP48 and WCFS downregulated MHC-IIβ (Figure 8). Another pathway that might contribute to the enhanced responses in TIFN101 is the upregulation of genes involved in leukocyte extravasation (Figure 9). TIFN101 enhanced RAPL expression, which is a GTPase involved in regulating integrin affinity. Concomitantly, an upregulation of essential adhesion molecules such as ICAM-1 and Cadherin 5 was seen, illustrating the upregulation of immune-cell migration pathways by TIFN101. Also, some regulation of leukocyte extravasation was observed with CIP48 and WCFS. However, it was much less pronounced than for the TIFN101 intervention.

### Differential Gene Content Profiles between the Three *L. plantarum* Strains

**Figure 10:**
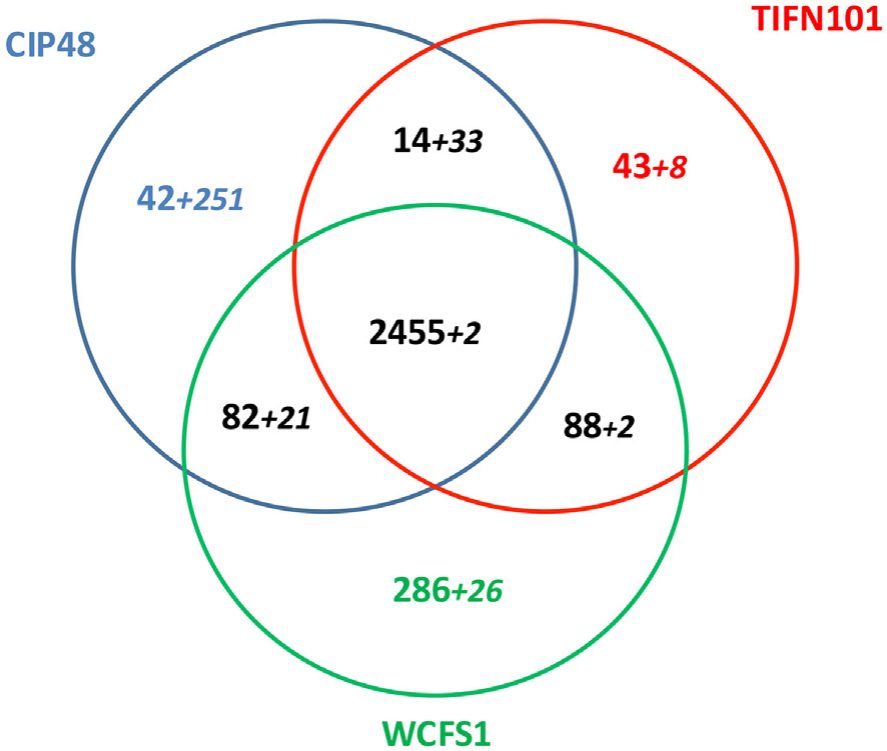
Venn diagram of shared and unique genes in all contigs of *Lactobacillus plantarum* strains [*L. plantarum* WCFS1 (WCFS), *L. plantarum* CIP104448 (CIP48), *L. plantarum* TIFN101]. The smaller numbers in italics represent ortholog groups that do not match to the chromosome of strain WCFS. These numbers do not include the putative prophage genes in each genome.

*Lactobacillus plantarum* strains CIP48 and TIFN101 were sequenced, annotated, and compared with the genome (chromosome and plasmids) of *L. plantarum* WCFS1 (35, 36). A total of 3,010 OGs were assigned to the chromosome, based on the ordering of contigs to the template WCFS genome. The three genomes shared 2,455 of the 3,010 chromosomal OGs (=81.5%), which is defined as the core genome for this study. Figure 10 presents the shared and genes and contigs of the *L. plantarum* strains. When the contigs and OGs/genes are included that do not match to the WCFS chromosome, higher numbers of unique genes were found for the CIP48 and TIFN101 genomes. Many of these extra unique genes are on plasmids (Table S2 in Supplementary Material). All unique genes for *L. plantarum* TIFN101 and *L. plantarum* CIP104448 compared to WCFS1 are listed in Tables S3 and S4 in Supplementary Material, and all absent genes in the two strains are listed in Tables S5 and S6 in Supplementary Material, respectively, as these are potential candidate genes for the biological effects of the two strains. *L. plantarum* CIP48 lacks the complete plantaricin biosynthesis gene cluster, a large set of genes adjacent to this cluster (i.e., OGs 334–348), and the entire gene cluster for EPS biosynthesis. *L. plantarum* TIFN101 is missing some genes associated with plantaricin biosynthesis as well as genes for exopolysaccharide biosynthesis, many sugar utilization cassettes, and two large LPXTG-anchored mucus-binding proteins (Tables S7A,B in Supplementary Material).

## Discussion

The current experimental human study was undertaken to investigate the immunomodulatory and potentially beneficial effects of three *L. plantarum* strains, which were selected *in vitro* for their differential immune stimulating capacity. Indicators of responses of mucosal and systemic immunity were assessed in healthy individuals undergoing a mild, commonly encountered stressor (25, 26) of the intestine and immune system, *i.e*., the intake of NSAIDs. We show that three *L. plantarum* strains had a highly strain-dependent effect on immunity *in vivo*, which were different to those predicted *in vitro*.

Indicators of systemic immunomodulatory effects were assessed in peripheral blood samples of the study participants. Consumption of indomethacin induced a reduction in CD4^+^/Foxp3 regulatory cell frequencies, which was prevented by WCFS and TIFN101 administration. This can be considered a beneficial regulatory effect. CIP48, not preventing the reduction of CD4^+^Foxp3 and reducing the number of memory cells, had effects that suggest a pro-inflammatory effect of consumption of this bacterium. Furthermore, T-cell polarization was studied after different stimuli. The hypothesis was that bacterial cell-wall components might induce immune responses (1) and enhance systemic immunity as a bystander effect. This happened with WCFS and TIFN101 (Figure 5). However, the most pronounced stimulator of immunity, i.e., TIFN101, also showed enhanced responses against specific pathogenic antigens such as TT and had an overall enhanced response to the SEB challenge. It is unlikely that the enhanced response against TT in the TIFN101 consumers is caused solely by the response against TIFN101 cell components. This is supported by the analysis of the intestinal biopsies that suggest that TIFN101 stimulate specific processes in the intestinal mucosa. TIFN101, in contrast to the other strains, upregulates mucosal transcription processes associated with T and B cell function and antigen presentation, when compared to placebo. It also had a pronounced effect on CD27 upregulation, which is required for generation and long-term maintenance of T cell immunity (37). Furthermore, TIFN101 enhanced expression of MHC-IIα in intestinal mucosa as well as key regulatory molecules such as RAPL. RAPL enhances integrin affinity and the adhesion of T cells (38, 39). These observations in the mucosa may explain the enhanced memory T cell responses observed with TIFN101 consumption. Furthermore, B cell immunity in the mucosa was enhanced as illustrated by upregulation of immunoglobulin regulatory genes and by CD79a. CD79a is also known as B cell antigen receptor complex-associated protein alpha-chain forming, together with CD79b protein, the B cell antigen receptor complex-associated protein (40). CIP48 and WCFS did not have these effects; in fact, they had a tendency to downregulate processes such as antigen presentation in the mucosa.

As a consequence of differences in TLR-binding, *L. plantarum* strains induce upon incubation with monocytes or dendritic cells different quantities of the pro-inflammatory cytokine IL-12 and the regulatory cytokine IL-10 (10). On the basis of these findings, three strains were selected, *L. plantarum* WCFS1 (WCFS), *L. plantarum* CIP104448 (CIP48), and *L. plantarum* TIFN101 (TIFN101), as their induced IL-10/IL-12 ratio *in vitro* were classified as pro-inflammatory, neutral, or anti-inflammatory, respectively. However, the immune responses to these strains *in vivo* were very different to that predicted *in vitro*. Consumption of NSAID induced a reduction in CD4^+^/Foxp3 regulatory cell frequencies, which was prevented by WCFS1 and TIFN101 administration. This should be considered to be a beneficial regulatory effect. CIP48 did not prevent NSAID-induced reduction of CD4^+^Foxp3 T cells and had more negative effects. CIP48 reduced the number of memory cells suggesting a pro-inflammatory, worsening effect of consumption of this bacterium. Our data suggest that solely studying the effects of bacteria on a single cell type *in vitro*, such as dendritic cells, has limited value, as it does not provide representative insight into the complex interplay between immune and other mucosal cells *in vivo*.

To our knowledge, this is the first report that bacteria can downregulate snoRNAs in the stressed intestine. SnoRNA are metabolically stable, non-coding RNAs that associate with a set of proteins to form small nucleolar ribonucleoproteins. The majority of snoRNAs function to guide RNAs in the post-transcriptional synthesis of 2′-O-methylated nucleotides and pseudo-uridines in ribosomal RNAs (rRNAs), small nuclear RNAs, and other cellular RNAs, including messenger RNAs (41–43). The relative reduction of several snoRNAs by TIFN101 and WCFS suggest a downregulation of methylation of rRNA (44) and downregulation of 14b diminished pseudo-uridinilation of RNA (45). Usually, this is indicative of a destabilization of cellular processes, but it may also be an indicator of tissue repair (46).

It should be noted that, in the present trial, we used a cross-over design to eliminate potential influences of inter-subject differences. Previous interventions may influence subsequent tests in such a study design. However, a long wash-out period of 4 weeks was chosen and the order of interventions was randomly assigned to each study subject, which should have minimized potential influences between interventions. Furthermore, the impact of *L. plantarum* consumption observed in this study may be caused by direct effects on host immunity; however, indirect effects *via* modulation of the resident microbiota may contribute as well.

Finally, full genome sequencing of *L. plantarum* CIP48 and *L. plantarum* TIFN101 was applied to identify possible gene clusters that might be responsible for the differential biological effects of the three *L. plantarum* strains. Several 100 novel *L. plantarum* genes were found in strain CIP48 and TIFN101 as compared to the known genome of strain WCFS (35, 36), of which only a small number are shared by both CIP48 and TIFN101. The majority of the novel genes appear to be located on plasmids. CIP48 appears to have several plasmids that are not present in TIFN101 or WCFS. Strain CIP48 has specific genes, such as for lantibiotic biosynthesis and several cell-surface proteins, that might explain its differential effect. Notably, however, there are also numerous genes/gene clusters in *L. plantarum* CIP48 and TIFN101 that are not present in the WCFS genome. Strikingly, CIP48 completely and TIFN101 partially lack the plantaricin biosynthesis clusters. These genes have been linked to strain differences in cytokine production (10, 47), but were shown here not to be associated with immune effects *in vivo*. Also, both strains lack very large regions important for sugar metabolism. These differences have been attributed to adaptations to environmental factors which, in our opinion, are interesting targets genes and possibly associated with probiotic effects (48). Not only the presence but also the absence of genes may enhance immune effects of bacteria (8).

## Conclusion

The current randomized double-blind placebo-controlled cross-over human trial demonstrated immune modulatory effects by orally consumed *L. plantarum* strains *via* specific responses against bacterial components as well as direct stimulation of specific immunity in the intestine. Specific bacteria can influence and possibly prevent undesired immune responses during commonly encountered stressors of the intestine such as intake of NSAIDs. Strong strain-dependent effects were found that showed *L. plantarum* TIFN101 to have a positive effect on host immunity, while CIP48 and WCFS1 had effects that may to be considered as less beneficial. Caution should be taken in selecting beneficial bacteria as even closely related strains can have very different effects. The observation that some bacteria can enhance specific memory cell populations can be helpful in identifying putative immune active human applicable bacterial strains. Furthermore, the current comparative genomics study provides many leads for follow-up experimental work to identify genes that are responsible for or involved in the observed differences in immune effects in humans.

## Ethics Statement

This study was carried out in accordance with the recommendations of the University Hospital Maastricht Ethical Committee with written informed consent from all subjects. All subjects gave written informed consent in accordance with the Declaration of Helsinki. The protocol was approved by the University Hospital Maastricht Ethical Committee. The study has been registered in the US National Library of Medicine (http://www.clinicaltrials.gov, NCT01456767).

## Author Contributions

PV co-supervised and co-designed the study and wrote the manuscript; ZM conducted the study and was involved in manuscript writing; BH and RS performed most analyses and reviewed the manuscript; PB provided the bacterial supplements and placebo; MM, JW, and AM co-supervised the study and interpretation of data; MB conducted the transcriptome analyses; and MF and FT co-supervised and co-designed the study.

## Conflict of Interest Statement

PB is employee of NIZO food research, a private commercial company. All other authors declare no conflict of interest.

## Footnotes

^1^http://www.clinicaltrials.gov.

^2^http://broad.mit.edu/gsea/.

^3^http://rast.nmpdr.org/.

^4^http://blast.ncbi.nlm.nih.gov/.

^5^http://www.ebi.ac.uk/interpro/.

^6^http://www.orthomcl.org/.
