# Supplementary material for: Lactobacillus plantarum Strains Can Enhance Human Mucosal and Systemic Immunity and Prevent Non-steroidal Anti-inflammatory Drug Induced Reduction in T Regulatory Cells
Source: Front Immunol. 2017 Aug 23;8:1000. doi: 10.3389/fimmu.2017.01000 (PMC5572349; doi:10.3389/fimmu.2017.01000)
Supplement: Supplementary file 1 [file Data_Sheet_1.pdf]

Table S1. Antibodies used for flow cytometry

Specificity	Clone Name	Fluorochrome	Supplier
CD3	UCHT1	Pacific Blue	BD Pharmingen
CD8	SK1	PerCP	BioLegend
CD69	FN50	PE-Cy7	BD Pharmingen
FoxP3	206D	APC	eBioscience
CD45RO	UCHL1	Biotin + streptavidin PerCP	BioLegend
IL4	MP4-25D2	PE-Cy7	BioLegend
IL-17A	eBio64DEC17	Alexa488	eBioscience
IL-21	eBio3A3-N2	PE	eBioscience
IFNγ	B27	Alexa700	BD Pharmingen
CD56	MEM-188	APC	eBioscience
CD16	CB16	EFluor450	eBioscience
CD335	9E2	PE	BioLegend
CD161	HP-3G10	PE-Cy7	eBioscience

Table S2. ORFs in prophage regions of *L. plantarum* TIFN101, *L. plantarum* CIP104448 (CIP48), and *L. plantarum* WCFS1 (WCFS1).

Prophage region	TIFN101	CIP48	WCFS1	Gene regions in WCFS1
I	0	46	61	lp_0624-0687
II	51	0	0	between lp_1162 and lp_1163
III	0	44	0	between lp_1363 and lp_1364
IV	70	17	74	lp_2399 -2480
V	4	0	0	between lp_2509-2512
VI	13	0	15	lp_3375-3391
**Total genes**	**138**	**107**	**150**	

Table S3. Summary of main unique contigs/gene clusters/genes in *L. plantarum* TIFN101

OGs	Contains functions	Comment
**Chromosome**		
185-187	Fumarate reductase/succinate dehydrogenase flavoprotein; MFS family transporter	
382-383	Toxin-antitoxin system MazE/MazF family	100% id to plantarum UCMA3037
409-411	Ankyrin repeat protein	Also in most plantarum genomes
461-463	Nitrate/sulfonate/bicarbonate ABC transporter	Also in most plantarum genomes
1197	ADP-ribosylglycohydrolase	Also in plantarum UCMA3037 and ZJ316
1986	Abortive infection bacteriophage resistance protein	
2366	Glycosyl hydrolase family 25	
2549	Membrane proteinase PrsW, regulator of anti-sigma factor	Not in other plantarum genomes
2583	Hypothetical membrane protein, YfhO family	
2610-2615	Fructose-specific PTS system	Also in most plantarum genomes
**On putative plasmids**		
3299-3308	Ribonucleotide reductase (aerobic); several transporters	99% id to plasmid pST-III of plantarum ST-III
3309-3322	EPS biosynthesis	Between transposases; could be on chromosome
3323-3327	Mannose-6-phosphate isomerase; glutamine synthetase	
3328-3334	toxin-antitoxin system MazE/MazF family; cold shock protein	99% id to plasmid p256 of plantarum NC7
3335-3347	Toxin-antitoxin system, RelE/StbE family; replication protein RepA	Definitely a plasmid
3348-3353	Pyridine nucleotide-disulfide oxidoreductase	99% id to plantarum UCMA3037
3354-3359	Oxalate:Formate antiporter; Excinuclease ABC subunit A paralog	
3360-3365	Peptidase E; alkaline shock protein	
3366-3371	Plasmid partitioning ATPase ParA; replication protein	Definitely a plasmid

Table S4. Summary of main unique contigs/gene clusters/genes in *L. plantarum* CIP104448

OGs	Contains functions	Comment
**Chromosome**		
228-229	N-acetylmuramoyl-L-alanine amidase	
293-297	Methionine ABC transporter	
762-766	Cell surface protein precursor, with SEC10/PgrA surface exclusion domain and LPXTG-motif cell-wall anchor	Also in plantarum ST-III
1073-1076	Esterase/lipase; transporter; regulators	Also in plantarum ZJ316 and JDM1
2328	Cell surface-exposed lipoprotein (phage super infection exclusion)	
2509-2519	Toxin-antitoxin system, RelE/RelF family; beta-glucosides PTS system; lactococcin A immunity protein; glycosyl hydrolase family 25; carboxymuconolactone decarboxylase	Also in most other plantarum genomes; also found on plantarum plasmids
2861-2862	Polysaccharide deacetylase, intercellular adhesion protein IcaB; N-glycosyltransferase, intercellular adhesion protein IcaA	No hits better than 40% id in NCBI
**On putative plasmids**		
3093-3098	Lantibiotic biosynthesis	Most similar to *Pediococcus claussenii* ATCC BAA-344
3099-3107	ATP-dependent endonuclease and DNA helicase; oligopeptidase F; transporters	
3108-3117	Type I RM system; DNA helicase	
3118	Cell surface protein precursor	94% id to *Lactobacillus fermentum* 28-3-CHN
3119-3126	D-Lactate dehydrogenase; glycogen biosynthesis	
3127-3134	Type I RM system;	
3135-3148	Toxin-antitoxin system; Polysaccharide deacetylase; mobilization proteins; replication protein; cation-transporting ATPase	definitely on a plasmid
3149-3159	Replication protein RepA; plasmid partitioning ATPase; MFS family transporter	definitely on a plasmid
3160-3165	Amino acid transporters	99% id to plantarum UCMA3037
3168-3175	Fumarate reductase flavoprotein; cation transporter	
3176-3184	Oxalate:Formate Antiporter; Nicotinamidase - Nicotinate phosphoribosyltransferase - transcriptional regulator NrtR	
3185-3189	Glycosyltransferase	
3190-3193	Cell-envelope associated proteinase PrtR	95% id to Enterococcus sp. GMD4E

3194-3199	Ferrochelatase; transporters	99% id to plantarum UCMA3037
3200-3205	Cysteine desulfurase NifS; Quinolinate synthetase; L-aspartate oxidase	Highest % id to Lactobacillus parafarraginis F0439
3206-3211	Heavy metal transporting ATPase	
3216-3218	Glycosyl hydrolase family 70	
3219-3220	Peptidoglycan hydrolase, GH25 family	
3224-3227	Abortive infection phage resistance protein; toxin-antitoxin system, MazE/MazF family	
3228-3232	Pyridine nucleotide-disulphide oxidoreductase; mobilization protein; cell-surface collagen-binding protein, LPXTG anchored	
3235-3236	Toxin-antitoxin system, MazE/MazF family	100% id to p256 of L.plantarum NC7
3246-3250	Plasmid partition protein ParA, ATPase; replication protein; amino acid transporter	Definitely on a plasmid
3251-3252	C-terminal processing peptidase	

Table S5. Summary of main contigs/gene clusters/genes absent only in *L. plantarum* TIFN101

OGs	Contains functions	Comment
**Chromosome**		
384-387	Beta-glucosides PTS system	
735-737	Beta-glucosides PTS system	
1218-1242.6	Iron chelatin ABC transporter; nitrate reductase and molybdenum cofactor biosynthesis	Whole cluster absent for nitrate assimilation
2331-2332	Prolyl aminopeptidase; proline-containing amino acid or peptide transporter	
2391-2393	Manganese transport protein MntH	Mn transporter is present on a plasmid in TIFN101
2477-2483	Oxidoreductases; regulators	
2501-2506	Elongation factor LepA; Na(+)/H(+) antiporter; fumarate reductase; regulators	
2530-2537	Glutamine amidotransferase; oxidoreductase; malate dehydrogenase; muramidase	
2811-2816	Shikimate metabolism	
2822-2827	Beta-glucosides PTS system; N-acetylmuramic acid-6-phosphate etherase; N-acetylglucosamine kinase	
**On putative plasmids**		
3025-3030	Arsenic resistance	On plasmid pWCFS103
3038-3055	Conjugation	On plasmid pWCFS103

Table S6: Summary of main contigs/gene clusters/genes absent only in *L. plantarum* CIP104448

OGs	Contains functions	Comment
**Chromosome**		
328	Extracellular protein, lysine-rich	
334-348	Transporters; regulators; hydrolases; many hypotheticals	
362-375	Plantaricin biosynthesis	CIP48 lacks the entire plantaricin gene cluster
991-995	Exopolysaccharide biosynthesis	CIP48 lacks the entire cps-cps2-cps3 biosynthesis cluster
1153-1158	Beta-glucosides PTS system	
2847-2854	Transaldolase, transketolase; PTS system (mannitol ?)	
2870-2878	Lipase/esterase; Na(+)/H(+) antiporter; N-acetylneuraminate transport and metabolism	
2896-2898	Pyruvate oxidases	
2954-2958	PTS system, glucitol/sorbitol-specific	

Table S7A. Summary of main novel contigs/gene clusters/genes present in *L. plantarum* CIP104448 and *L. plantarum* TIFN101, but absent in *L. plantarum* WCFS1.

OGs	Contains functions	Comment
**Chromosome**		
150	Alpha-galactosidase	
1183	Niboflavin biosynthesis protein RibD	also in other plantarum genomes; WCFS1 is known to have a disrupted riboflavin biosynthesis gene cluster
1488-1489	Niacin transporter NiaP; Quinolinate phosphoribosyltransferase [decarboxylating]	highest identity to *L. helveticus*
**On putative plasmids**		
3400-3409	Arabinose efflux permease; Polysaccharide pyruvyl transferase; sugar/inositol transporter; Pyruvate oxidase	
3064-3066	Trehalose 6-phosphate phosphorylase; Beta-phosphoglucomutase; HAD superfamily hydrolase	
3067-3071	Multicopper oxidase CueO; multidrug transporter	
3077-3086	Manganese transport protein MntH and regulator MntR; Kup system potassium uptake protein	MntH is present on chromosome of WCFS1
3087-3092	Polar amino acid ABC transporter	

Table S7B. Summary of main unique contigs/gene clusters/genes absent in both *L. plantarum* CIP104448 and *L. plantarum* TIFN101

OGs	Contains functions	Comment
**Chromosome**		
201-208	Sugar PTS system	
349-361	Plantaricin biosynthesis	CIP48 lacks the entire plantaricin gene cluster, while TIFN101 lacks plnRLKJMNOPQ genes
971-990<br>998-1026	EPS biosynthesis	CIP48 lacks the entire cps-cps2-cps3 biosynthesis cluster, whereas TIFN101 only has 3 genes (which could be on a plasmid instead)
2075-2081	Formate metabolism; regulators	
2113-2117	PTS system, N-acetylglucosamine-specific	
2419-2430	Oxidoreductases	
2485-2500	iron chelatin ABC transporter; oxidoreductases;<br>mucus-binding protein, LPXTG-anchored; cell surface complex CscABC	
2508	Mucus-binding protein, LPXTG-anchored	
2520-2529	PTS system, beta-glucosides-specific; transketolase; sugar permease	
2539-2548	Bile acid 7-alpha-dehydratase; oxidoreductase; transporter	
2603-2607	PTS system, beta-glucosides-specific	
2828-2846	Sugar PTS systems	
2863-2869	Arabinose uptake and metabolism	
2892-2893	Auto-inducing peptide (AIP) and its export/modification	
2899-2953	Many sugar utilization gene cassettes (rhamnose, galactitol, myo-inositol, glucitol/sorbitol, mannose-related oligosaccharides)	
